# MALAT1 and HIST1H2BC as connecting diagnostic biomarkers in Ebola and its overlapping disease: An integrative approach

**DOI:** 10.6026/973206300212878

**Published:** 2025-08-31

**Authors:** Shaheen Hayat, Khalid Raza, Mohd Murshad Ahmad, Rafat Ali, Naaila Tamkeen, Akhtar Veg, Najma Khan, Faria Saleem, Romana Ishrat

**Affiliations:** 1 Centre for Interdisciplinary Research in Basic Sciences, Jamia Millia Islamia, New Delhi, India; 2 Department of Computer Science, Jamia Millia Islamia, New Delhi, India; 3 Department of Biosciences, Jamia Millia Islamia, New Delhi, India

**Keywords:** Ebola virus disease, connecting diagnostic biomarker, differentially expressed gens, overlapping disease, network biology

## Abstract

Diagnostic biomarker for Ebola Virus Disease (EVD) and its overlapping diseases, which include COVID-19, monkey pox, AIDS and uveitis, is of interest. The gene-disease association network was built by using human microarray datasets from the GEO database. The comparison based on Jaccard's similarity index showed 27 intersected differentially expressed genes (DEGs), with AIDS having the highest genomic association with EVD. Gene-disease interaction analysis revealed Metastasis-associated lung adenocarcinoma transcript-1 (MALAT1) and histone cluster 1, H2bc (HIST1H2BC) as the most relevant diagnostic biomarkers. Thus, the dg IDB database provided potential for drug repurposing, targeted therapy and broad-spectrum antiviral applications.

## Background:

The infection of the Ebola virus leads to an acute and life-threatening condition in humans, which is marked by hemorrhagic fever, coagulation or bleeding, an increased inflammatory reaction, hypotensive shock and frequently, fatality [1]. The Ebola virus belongs to the Filoviridae family and comprises six virus species within the Ebolavirus genus, which are Zaire, Sudan, Tai Forest (formerly known as Cote d'Ivoire), Reston, Bundibugyo and Zaire ebola virus Bombali [2, 3]. Of the six identified species, Zaire ebolavirus shows the highest rates of case fatality in human populations. Zaire ebola virus was responsible for the Ebola virus outbreak that occurred in West Africa from 2014 to 2016 and this epidemic stands as the most extensive in history, encompassing in excess of 28,000 documented cases and a death toll exceeding 11,000 individuals [4]. Apart from the outbreak in Western Africa, the outbreak in the provinces of Ituri, Nord-Kivu and Sud-Kivu in the Democratic Republic of the Congo, is the second largest outbreak in terms of the number of cases and fatalities, with 3,418 infections and 2240 deaths at the time of 2018- 2020 [5, 6]. The present literature suggests that fever, muscle-joint pain, fatigue, uveitis eye inflammation and respiratory difficulties, are the most commonly reported symptoms associated with EVD, COVID-19, AIDS and Monkey Pox (MPOX) viral diseases. Inflammation is a prominent characteristic that arises during or subsequent to various viral diseases, including but not limited to EVD, COVID-19, MPOX and AIDS. There appears to be a significant correlation between the ocular system and the complications of immune-mediated or systemic diseases. Due to its immune-privileged nature, the Uvea, which is one of the most delicate parts of the human eye, is particularly susceptible to direct infection or immune-mediated complications. This vulnerability is due to the eye's high level of sensitivity, making it a crucial organ in the human body. Uveitis can lead to enduring morbidity and reduced quality of life for patients. Nevertheless, the intricate interaction between EVD and different ODs remains incompletely comprehended [7, 8, 9, 10, 11, 12, 13-14]. Overlapping diseases are medical conditions which coexist and share clinical features/symptoms of at least two or more widely recognised diseases [15]. The genetic correlation between two diseases or infections is established when they share common dysregulated genes [16]. The study of gene-disease interaction, specifically in the context of infectome or diseasome network, facilitates the identification of novel interactions that can elucidate the genomic association or molecular mechanisms of correlated viral diseases [17]. The presence of ODs has been found to significantly increase the risk of morbidity and mortality due to associated infections and diseases [18]. It is obvious that the epidemiological transition results in a double disease load in the affected population and is becoming an important health concern globally. The commonality of EVD and its ODs raises pharmacological concerns and presents a significant barrier for co-management and treatment, indicating the need for an innovative shift, highlighting common biomarkers and treatment targets rather than stand-alone approaches emphasized on particular and discrete diseases.

In a recent study, Gysi *et al.* used a network-medicine and drug-repurposing strategy to find potential re-pursuable drugs for the treatment of COVID-19 [19]. In their study, Sakle *et al.* implemented a network pharmacology-based methodology to demonstrate the multifaceted pharmacological properties of Caesalpinia pulcherima (CP) in the context of breast cancer treatment. The findings of their investigation provide valuable insights into the potential clinical applications of CP as a multi-target herb [20]. Azuaje, *et al.* have contributed to the understanding of the cardiovascular impacts of non-cardiovascular medications through the integration of various drug and protein interaction data sources to construct the drug-target interactome network for myocardial infarction [21]. Zhu *et al.* suggested that Gene-Disease interaction within a network offers a novel perspective to find molecular biomarkers for comorbidity of viral infections [22]. Omit *et al.* implemented a network based Gene-Disease interaction approach to discover the genomic associations and molecular mechanisms for COVID-19 associated diseases [23]. Kim *et al.* proposed that the proximity between drugs and diseases within a network could provide a unique approach to understanding the therapeutic combination therapies and drug repositioning strategies [24]. The theoretical framework and methodological tools of network analysis are ideal for investigating and comprehending the structural and relational dimensions of human health and diseases [25]. The application of network-based analyses is becoming increasingly significant in identifying genes associated with disease susceptibility and their correlations with various diseases. The aforementioned studies have contributed to the advancement of our knowledge regarding drug targets and their impacts. Additionally, they have proposed novel drug targets, therapeutics and therapeutic management strategies for severe illnesses [26]. The study of networks is playing an important part in the advancement of systems pharmacology. The potential existence of mechanisms and a causal relationship between EVD and the ODs requires further substantiation. In order to gain a more comprehensive understanding of the underlying mechanisms, the present research was proposed to construct a gene-disease interaction network (bipartite graph) by using the intersecting of EVD with other ODs. The gene-disease network has been examined to find out the EVD related genes that are also associated with chosen ODs and to establish the relationship between genes and diseases, in which a minimum of one gene that is significantly up regulated or down-regulated should be common in EVD and its ODs, or within the ODs. Next, the Jaccard similarity index was used to determine the predominant overlapping disease within the chosen set of ODs. Protein-protein interaction network of EVD and ODs was also build using differentially expressed genes of EVD and ODs [27]. In order to assess the molecular mechanisms, we performed structural and functional enrichment and pathway analysis. Ten biomarker hub genes were identified based on their degree of influence, as elaborated in the methods and analyses section. Therefore, it is of interest to describe novel findings that shed further light on the biological basis of therapeutic responses and the development of targeted and multidrug treatments for EVD and its ODs.

## Methodology:

The simplified workflow of this study is shown in Figure 1 (see PDF).

## The selection process of microarray datasets related to EVD and its ODs:

The GEO (Gene Expression Omnibus) from NCBI is a publicly available repository that comprises gene expression profiles [28]. The study utilised five microarray datasets, namely GSE93861 [29], GSE150819 [30], GSE9927 [31], GSE36854 [32] and GSE66936 [33] for EVD, COVID-19, AIDS, MPOX and Uveitis respectively which were obtained from the GEO datasets repository [34]. The datasets utilised in our study were chosen through a rigorous process of inclusion and exclusion criteria, which specifically targeted (i) studies involving both human subjects diagnosed with disease and healthy control groups. (ii) Assessment of expression of genes profiling. (iii) The inclusion criteria for studies involve selecting those with a minimum of six control and six experimental samples. (iv) The datasets were excluded if the studies did not include a healthy control group. (v) The datasets from other organisms were omitted. All datasets and references that met the aforementioned criteria underwent manual screening. As this study solely relies on bioinformatics analysis, ethical approval was considered unnecessary.

## Finding of differentially expressed genes of EVD and its ODs:

The identification of DEGs was carried out through a comparison between normal and disease samples within each GEO dataset. The DEGs were found via the utilisation of the online programme GEO2R [35], which relies on the limma R package [36]. The main cut off parameters selected for interpreting the results were a "P value < 0.05" and "|logFC|≥1". Important DEGs were identified for each category, namely EVD, COVID-19, AIDS, MPOX and Uveitis, by applying a cut-off criterion to each dataset. Only those DEGs that met the cut-off criteria in each dataset were included in the analysis. The list of interconnecting DEGs was obtained through utilisation of Venny 2.1.0, an online computational tool that facilitates the calculation of intersections among listed elements (Figure 2 - see PDF).

## EVD and its ODs linkage:

By employing community prospered standard and topological methodologies, we developed bipartite networks or graphs to represent the relationship between genes and diseases. The nodes in the network were either genes (represented by round shapes) or diseases (represented by octagonal shapes). Through this approach, we were able to confirm the correlation between EVD and its ODs. In order to take part in the network and establish an association or connection, it is necessary for the diseases of interest, namely EVD and its ODs, to have one or more significant DEGs in common. The present study involved the consideration of M as a set of maladies and D as a set of DEGs. The bipartite graph or network was established based on the affiliation of gene g E D with malady m E M. If DEGs D_A_ and D_B_ exhibit a gradual correlation with maladies M_A_ and M_B_, respectively, the duplicated shared DEGs (see PDF) for both upregulated and down-regulated genes in the maladies can be expressed mathematically as stated below: (see PDF)

[1] The identification of common close neighbours and their interactions are achieved by means of the computation of the edge score (E) for every pair of nodes, using the Jaccard's similarity index [37, 38]. (see PDF)

[2] The sets D and E represent the nodes and edges, respectively. In the context of networks or bipartite graphs, co-occurrence refers to the quantity of genes that are shared.

## Protein-Protein interaction network construction of EVD and its ODs:

The study analysed the interaction between DEGs at the protein level using protein-protein interaction data from the STRING database [39]. The PPI network included 150 nodes in the 1st and 2nd shell interactors, ensuring a greater number of seed genes were included. Cytoscape V3.10.2 [40] was used for visualizing the network. The properties mentioned below were analysed to find out significant characteristic of the constructed PPI network:

[1] Degree distribution: The degree distribution is the probability of a randomly selected node having a specific degree, expressed as a proportion of the total network node count [41]. (see PDF)

[2] Clustering co-efficient: The Clustering coefficient C(k) measures the inherent clustering tendency of nodes in a network, assessing the strength of internal connectivity among nodes' neighbourhoods. It is calculated by comparing the number of triangular motifs in the network [42-43]. (see PDF)

[3] Neighbourhood connectivity: The average connectivity determined by the nearest neighbours of a node with degree k is denoted by C_N (k), which is also known as the node neighbourhood connectivity [44]. The mathematical expression of is as follows: (see PDF)

Where, P (q k) is the conditional probability.

[4] Betweenness centrality: (CB (v)) is the proportion of shortest-path flow from nodes i to j, indicating a node's potential for network information dissemination and signal processing regulation [45]. (see PDF)

[5] Closeness centrality: Closeness centrality (CC) measures the shortest path distances between a node and all other connected nodes, indicating the rate of information spread in a network. The clustering coefficient (C_C) is determined by dividing the total number of nodes by the total geodesic path lengths [46]. (see PDF)

## Identification and analysis of sub-networks/modules and hub genes:

The study used the MCODE plug-in [47] in Cytoscape software to identify subnetwork/modules, with clustering parameters such as degree cut-off, node score, k-core value and maximum depth. Top 5 modules were selected based on cluster scores greater than 6 and Centi Sca Pe [48] was used to find inter modular hub genes. From each module only top 10 genes having highest score on the basis of their degree centrality were picked for the purpose of identifying significant and strong candidate genes. Furthermore, we calculated hub genes in the primary network through cytohubba [49].

## The functional enrichment and pathway analysis:

The present study employed The Database for Annotation, Visualisation and Integrated Discovery (DAVID) and the g-Profiler tool to derive biological significance from extensive gene lists [50, 51]. This was done to conduct comprehensive gene term enrichment and pathway enrichment analysis of the target genes [52]. Statistically significant functions and pathways were chosen based on an adjusted Benjamini P-value threshold of less than 0.01.

## Overlapping diseases interlinkage analysis:

The interconnections among the chosen ODs were also investigated. In this study, we looked over the dysregulated genes or DEGs of ODs associated with EVD. The identification of overlapped linking DEGs was carried out by applying Equations (2) and (3). Additionally, the gene-ODs interconnection network was constructed among the ODs themselves. Furthermore, a comprehensive search was conducted on globally published scientific literature to investigate the interrelationships between the chosen ODs and their impact on the progression and severity of EVD.

## Drug-target interactions:

In order to ascertain the interactions between drugs and their targets, we incorporated the DGIdb database [53] (www.dgidb.org). In this study, a total of 27 genes that serve as linking genes were subsequently mapped onto the Drug- Gene Interaction database (DGIdb). The purpose of the mapping was to ascertain more effective pharmacological options for genes associated with EVD and its ODs. The Cytoscape software was utilised to visualise the complex of drug-gene interactions.

## Results:

Table 1 (see PDF) presents the detail of the datasets related to the microarray series utilised in this research. Significant differentially expressed genes (DEGs) were identified based on satisfying the cut-off criteria of "P-value < 0.05" and "|logFC|≥ 1" in every single series. Only DEGs satisfying these criteria were included in the analysis. The adjusted p-value for various analyses was not utilised in our study. Instead, we employed a p-value cut-off of less than 0.05 to identify the largest number of differentially expressed genes (DEGs) associated with EVD and overlapping diseases. Subsequently, we proceeded with the construction of the network. The study identified a total of 1454 genes that exhibited differential expression in EVD. Among these genes, 681 were observed to be upregulated while 773 were observed to be down-regulated. In the case of COVID-19, we discovered 1396 DEGs, 831 of which were upregulated and 565 of which were down-regulated. We obtained a total of 947 DEGs for MPOX, of which 472 exhibited upregulation and the remaining 475 indicated down regulation. We found 1024 DEGs in AIDS, of which 432 were upregulated and 592 were down-regulated. Similarly, 797 genes were differentially expressed in Uveitis, including 383 upregulated DEGs and 414 down-regulated DEGs. The above-mentioned genes were utilised in the construction of the protein-protein interaction (PPI) network, which facilitated a comprehensive understanding of the intermolecular interactions between proteins in EVD and its overlapping diseases. Table 2 (see PDF) shows a summary of the DEGs. In order to establish a relationship, it is necessary for there to be at least one shared gene between the two illnesses.

Cross-comparative analyses were conducted to find shared DEGs between EVD and chosen ODs. The shared DEGs were determined to have a direct effect on the severity of EVD. Our study revealed that EVD has a total of 78 DEGs (53 upregulated and 25 down-regulated) that are shared with COVID-19, 64 DEGs (12 upregulated and 52 down-regulated) that are shared with MPOX, 87 DEGs (24 upregulated and 63 down-regulated) that are shared with AIDS and 53 DEGs (23 upregulated and 30 down-regulated) that are shared with Uveitis. Figure 3(a) (see PDF) demonstrates a total of 47 adjacent DEGs that are upregulated and shared exclusively between EVD and COVID-19. These DEGs are in close proximity to each other. There are an additional 6 DEGs that are commonly shared between EVD and COVID-19, along with other ODs. Likewise, there exists a set of 12 closely adjacent DEGs between MPOX and EVD. There are 19 DEGs that are commonly shared between AIDS and EVD. Additionally, there are 5 DEGs that are shared between EVD, AIDS and other ODs. The study demonstrated that there are 18 DEGs that are located next to each other in both EVD and Uveitis. Additionally, there are 5 DEGs that are located in between Uveitis, EVD and other conditions. Among the down-regulated shared DEGs, there are 19 adjacent DEGs that are exclusively common between COVID-19 and EVD. Additionally, 6 DEGs are found between EVD and other conditions, including COVID-19. There are 43 DEGs that are shared between MPOX and EVD. Additionally, there are 9 more DEGs that are common between EVD, MPOX and other ODs, as illustrated in Figure 3(b) (see PDF). There are 49 DEGs that are shared between EVD and AIDS and an additional 14 DEGs that are shared between AIDS and other conditions associated with EVD. There are a total of 20 DEGs that are located next to each other and are common to both EVD and Uveitis. Additionally, there are 10 other DEGs that are common to EVD, Uveitis and other conditions.

The Jaccard similarity index was determined using the DEGs of EVD and ODs. The resulting values were 0.028 for EVD and COVID-19, 0.027 for EVD and MPOX, 0.033 for EVD and AIDS and 0.024 for EVD and Uveitis. Neighbourhood similarity (Jaccard's similarity index) is a viable metrics for quantifying the interaction between two nodes [54]. There is a positive correlation between the neighbourhood similarity index of adjacent nodes and the level of interaction between the two nodes [55]. Out of the four ODs, AIDS demonstrated the highest similarity score. Table 3 (see PDF) presents the computation of the Jaccard similarity index, which utilises the DEGs associated with EVD and its ODs. Figure 2 (see PDF) displays the EVD and ODs association networks in order to demonstrate their vital relationship. The networks utilise frequent up and down-regulated DEGs to establish a connection between the selected ODs and EVD. The construction of the protein-protein interaction network involved the utilisation of overlapping DEGs that were shared among COVID-19, MPOX, AIDS and Uveitis. As a result, to make this network we took a total of 282 DEGs. Our objective was to create a network that includes the majority of the shared genes within the same network while ensuring that the network's clustering coefficient is greater than 0.5, a clustering coefficient greater than 0.5 indicates that the network and its genes are tightly clustered together. The primary network comprised of 606 nodes and 16,346 edges. In this context, the nodes represent proteins and the edges represent their interactions. Protein-protein interactions can be effectively studied by representing them as networks using graph theory. Subsequently, the topological properties of the network were determined.

The study found that the degree distributions P (k), average clustering coefficient C (k) and neighbourhood connectivity CN (k) demonstrate the fractal characteristics of the network. This indicates that the network possesses a self-organizing property, whereby the nodes retain their nature at different levels, rather than adhering to a centrality-lethality control system, meaning that removing one or more hubs does not result in network breakdown. The analysis of the network behaviour revealed that it complied with a hierarchical scale-free network, with all of its topological properties conforming to power-law distributions [56]. The standard statistical fitting method proposed by Clauset *et al.* was utilised to execute power-law fitting on the data points pertaining to the topological properties [57]. The negative values observed for P (k) and C (k) suggest that the network adheres to a hierarchical pattern. However, the positive value of CN (k) indicates that the network exhibits assortative mixing, which recognises the presence of clusters or "rich clubs" that regulate the network. The utilisation of network centrality measurements, specifically CB (k) and CC (k), serves to illustrate the propagation of information within a network and predict the nodes that hold the greatest influence (Figure 4 - see PDF). The PPI network consisting of seed genes was analysed using MCODE, resulting in the generation of 15 subnetworks or modules. Among the 15 modules, 10 required a degree cut-off value of 2 or higher, while 5 modules required a cluster cut-off score of 6 or higher. A total of 184 genes were identified in the top 5 subnetworks/modules, which were generated based on the density of the interactions. Next, we attempted to pare down a selection of inter-modular hub genes by utilising the degree centrality method. This involved submitting the 606 genes to the CentiScaPe plugin. The five most significant modules (Refer to Figure 5 - see PDF) contained a total of ten hub genes within each module. These hub genes were selected based on their highest degree centrality within their respective modules. Furthermore, through assessment of the PPI network that was obtained from the intersecting DEGs, we have successfully pinpointed 10 significant hub genes. This was accomplished by utilising the cytohubba plug-in and assessing the degree centrality of each gene. The hub genes that we have found are ranked based on their degrees, with TP53 having the highest degree of 159, followed by RPS27A with a degree of 148, MYC with a degree of 144, HSP90AA1 with a degree of 114, CDK2 with a degree of 111, ATM with a degree of 110, EP300 with a degree of 110, CCNA2 with a degree of 109, CCNB1 with a degree of 108 and CCND1 with a degree of 103. Using the DAVID functional annotation tool and g-Profiler tool, we found numerous molecular functions, biological processes and cellular components, as well as KEGG pathways [58], in which the candidate genes for EVD and ODs are considerably enriched, as shown in figure. By analysing the MF, the majority of the candidate genes were identified to be enriched in ubiquitin protein ligase binding, activating transcription factor binding, cyclin-dependent protein serine/threonine kinase regulator activity, p53 binding, protein kinase regulator activity, structural constituent of ribosome and ubiquitin-like protein ligase binding. For BP analysis, the maximum no. of genes were enriched in cell cycle G1/S phase transition, G1/S transition of the mitotic cell cycle, positive regulation of cell cycle, response to decreased oxygen levels, response to oxygen levels. In contrast, the CC analysis provided the majority of genes that were enriched in chromosomal region, cyclin-dependent protein kinase holoenzyme complex, cytosolic ribosome, protein kinase complex, ribosomal subunit, ribosome serine/threonine protein kinase complex, spindle transferase complex and transferring phosphorus-containing group. For pathway enrichment analysis, most genes were enriched in Cell cycle, Cellular senescence, colorectal cancer, Gastric cancer, Hepatocellular carcinoma, Human papillomavirus infection, Human T-cell leukemia virus 1 infection, Prostate cancer and viral carcinogenesis. These functions and pathways could play critical roles in the aetiology of EVD and its ODs (Figure 6 - see PDF). It has been observed that our ODs are finally interconnected through the exchange of linking DEGs among themselves. In addition, the international publications document the interaction, evolution and development of these linking DEGs. The study revealed that AIDS, COVID-19 and Uveitis had 1 DEG in common. AIDS, MPOX and Uveitis also had 1 DEG in common, while AIDS and COVID-19 had 8 DEGs in common. COVID-19 and Uveitis had 3 DEGs in common. On the contrary MPOX and Uveitis had 5 DEGs in common. Additionally, AIDS and Uveitis shared 5 DEGs, while AIDS and MPOX shared 3 DEGs. The interaction among the ODs is illustrated in Figure 7 (see PDF) through an association network. The findings indicated that the majority of the linking genes identified their targets, with the exception of some genes. Figure 8 (see PDF) depicts the number of drugs that interact with specific target genes. The findings indicate that genes that exhibit a greater level of interaction with multiple drugs may be more closely associated with the fundamental mechanisms that underlie the pathological phenotype associated with these drugs. Refer to Supplementary for a comprehensive list of target genes and their drugs.

## Discussion:

This study revealed two significant genes, MALAT1 and HIST1H2BC, as connecting novel diagnostic biomarkers of EVD and its ODs. The MALAT1, known as the metastasis-associated lung adenocarcinoma transcript 1, is a long non-coding RNA (lncRNA) that plays a crucial role in regulating gene expression. Its dysregulated expression has been linked to the pathogenesis and advancement of various malignancies in humans [59]. The primary localization of MALAT1 is within nuclear speckles, which are sub-nuclear regions known for their high dynamism and involvement in the storage, alterations and/or assembling of splicing factors [60]. Previous study suggested that the role of MALAT1 lies in its ability to enhance the stability of the interaction between the poly-pyrimidine tract-binding protein 1 (PTBP1, also referred to as hnRNP I) and PTB-associated Splicing Factor (PSF). This interaction forms a functional module that plays a crucial role in the regulation of pre mRNA alternative splicing (AS). Furthermore, it has been suggested that this functional module may have implications in the development and progression of hepatocellular carcinoma [61]. Another study demonstrated that Malat1 plays a pivotal role in the pathogenesis linked to deviant macrophage activation, thereby establishing Malat1 as a promising therapeutic target for ameliorating this cohort of disorders [62]. The hypothesis that long non-coding RNAs (lncRNAs) regulate transcription through chromatin modification has gained substantial support from numerous research conducted in the past decade. This mechanism of lncRNA regulation is believed to play a crucial role in the antiviral response [63]. As an illustration, the lncRNA MALAT1, which is triggered by HIV-1, effectively directs the core constituent of PRC2, EZH2, away from the promoter region of HIV-1's long terminal repeat (LTR). This phenomenon results in the dissociation of PRC2, which is responsible for the deposition of H3K27me3, thereby mitigating the epigenetic suppression of HIV-1 gene expression. The reactivation of transcription promoted by the long terminal repeat (LTR) of HIV-1 is a critical process necessary for both viral replication and the establishment of latency [64]. Considering the empirical evidence that individuals diagnosed with non-small cell lung cancer (NSCLC) demonstrate a heightened prevalence of Covid-19, coupled with the manifestation of more severe symptoms and unfavourable prognoses, it is plausible to assert that the elevated expression of MALAT1 in NSCLC patients may impede the innate antiviral defence mechanisms, thereby augmenting their vulnerability to SARS-CoV-2 infection. Nevertheless, additional research is imperative to elucidate the precise function of MALAT1 in the context of SARS-CoV-2 [65]. The function of MALAT1 in inhibiting apoptosis of myocardial cells has been established by its ability to reduce the expression of the PTEN (phosphatase and tensin homolog) gene via the action of miR-320 [66]. It is noteworthy that the upregulation of MALAT1 was identified in response to the aforementioned associated disorders. Numerous investigations have substantiated the upregulation of MALAT1, a long non-coding RNA, in various cancerous tissues. This overexpression has been linked to heightened metastatic activity and unfavourable prognoses in lung cancer [67], breast cancer [68], colon cancer [68], esophageal cancer [70] and several other malignancies. MALAT1 has additionally been implicated in the regulation of histone acetylation [71], the process of endothelial-to-mesenchymal transition (EMT) [72]. Recent studies have brought attention to an additional function of MALAT1 lncRNA in viral infection and innate immune responses, further confirming its significant involvement in several biological processes. The study conducted by Wei *et al.* examines the involvement of MALAT1 in inflammatory damage subsequent to lung transplantation, potentially providing valuable insights into the role of MALAT1 in inflammation-induced injury following SARS-Cov-2 infection. It is noteworthy that the suppression of MALAT1 expression resulted in the mitigation of inflammatory damage through the inhibition of neutrophil chemotaxis and the influx of immune cells to the infection site [73]. The authors propose that this mechanism may regulate the development of acute lung injury via the NF-kB and p38 MAPK pathways [74]. Additionally, it is feasible that the inhibition of neutrophil chemotaxis may alleviate the severity of cytokine storms in lung inflammatory injury. In their study, Bhattacharyya *et al.* elucidate the functional significance of MALAT1 in the context of two flavi-viruses, namely Japanese encephalitis virus (JEV) and West Nile virus (WNV).The Neuro2a cells, upon treatment with these viruses, exhibit and upregulation of MALAT1, which subsequently enhances the inflammatory response [75]. MALAT1, in conjunction with lncRNA NEAT1, has demonstrated potential as biomarkers for HIV infection, following the identification of elevated amounts of these long non-coding RNAs in peripheral blood mononuclear cells (PBMCs) after infection [76].

HIST1H2BC belongs to the histone H2B family. Histone modification appears to play a role in tumorigenicity, with mutations in histone H2B identified as major cancer drivers [77, 78]. It was reported that the HIST1H2BC gene had the greatest disparity in expression between brain and lymph node metastases in individuals diagnosed with metastatic breast cancer [79]. However, few studies have examined its relationship with cancer prognosis, which needs additional exploration. A strong correlation was discovered between the prognosis of bladder cancer and HIST1H2BC gene. HIST1H2BC was found to be specifically expressed in neutrophils. This gene contributes to the unique transcriptional profile of neutrophils, which helps in identifying neutrophils within the tumour microenvironment [80]. Previous studies have demonstrated that the expression of Hist1h2bc is increased in the ageing retina [81]. Another study showed that a reduction in the protein expression of HIST1H2BC, which possesses antibacterial properties, can enhance the capacity of TNF-alpha (gene: TNF) to induce the upregulation of genes linked with inflammation. Hence, it is plausible that mutations occurring in these genes may contribute to the heightened inflammatory infiltrate reported in the subset characterised by high CD8 T cell count, as well as in the subset exhibiting an abundance of both T cell types [82, 83]. It has been reported that HIST1H2BC is associated with human papilloma virus infection [84]. The infection with Pseudomonas aeruginosa bacillus resulted in the downregulation of the HIST1H2BC gene [85]. Although several studies have been done on biomarkers identification, still there is a void of research where common biomarkers were useful in diagnostic purpose or therapeutic management of similar type of viral disease. However a particular study based on biomarker identification associated with viral comorbidities shows that chikungunya has relation with Ebola Virus, Dengue and Semliki Forest Virus characterized by inflammations in these viral diseases and it aimed to discover common biomarkers for comorbidity of viral infections. They built relationship networks based on the Chikungunya virus after identifying shared genes among the illnesses mentioned above. Their analysis revealed that 1 gene KCNMA1 is commonly dysregulated among Chikungunya virus, Ebola virus and pain, 1 gene CELF4 is commonly dysregulated among Chikungunya virus, Ebola and Semliki Forest virus and GOS2 is common among Chikungunya virus, Ebola virus, Dengue and pain, another 1 gene ARNTL2 is common among Chikungunya virus, Dengue, Semliki Forest virus and pain. However 2 genes (B3GNT9 and BCL2L11) play an important role and differentially expressed among Chikungunya virus, Ebola virus, Semliki forest virus and pain. According to this study, the identified key genes MALAT1 and HIST1H2BC may serve as potential connecting biomarkers and therapeutic targets for EVD and its ODs in the future. There are certain limitations to consider, one of which is the limited sample size. At now, the existing datasets do not provide enough information for conducting a comprehensive investigation. Therefore, increasing the sample size would yield a more comprehensive outcome. Additionally, the ODs examined in this study are diverse in nature and it is possible that there are other factors that could also contribute to their occurrence. Despite its limitations, this analysis has the potential to yield more accurate results based on an analysis that combines integrated network biology and bioinformatics methodologies.

## Conclusion:

This study identified MALAT1 and HIST1H2BC as novel diagnostic biomarkers and 27 shared differentially expressed genes (DEGs) common to Ebola Virus Disease (EVD) and its overlapping diseases. Understanding the functions of these genes may lead to improved diagnostics and therapies for individuals affected by these conditions. Further external validation studies are crucial to confirm these findings and assess the impact of these genes on disease progression.
